# Wider stakeholders’ perspectives on successfully implementing electronic patient-reported outcome systems across organisations: a qualitative study in kidney care

**DOI:** 10.1186/s41687-026-01137-7

**Published:** 2026-07-15

**Authors:** Sabine N. van der Veer, Angelo Ercia, Fergus J. Caskey, Ken Farrington, Michael Rees, Sarah Knowles

**Affiliations:** 1 Division of Informatics, Imaging and Data Science, Manchester Academic Health Science Centre, University of Manchester, Manchester, UK; 2 Evergreen Life, Manchester, UK; 3 Population Health Sciences, Bristol Medical School, University of Bristol, Bristol, UK; 4 Renal Unit, East & North Hertfordshire NHS Trust, Stevenage, UK; 5 School of Social Sciences, Nottingham Trent University, Nottingham, UK

**Keywords:** Health information systems, Health services administration, Kidney diseases / therapy, Patient reported outcome measures, Qualitative research

## Abstract

**Background:**

Large-scale ePRO systems inherently need to cross organisational boundaries to meet their full potential for improving care and outcomes. Doing this successfully requires involving all stakeholders. However, previous research in kidney disease and other conditions mainly focused on individual patients and healthcare professionals, leaving wider stakeholders’ perspectives on how to implement cross-organisational ePRO systems unknown.

**Methodology:**

We conducted 18 semi-structured interviews with key informants from national stakeholder organisations involved in delivering, commissioning, evaluating, or improving hospital-based kidney services in the United Kingdom. Interviews explored participants’ views on kidney ePRO systems, including their perceived value, implementation challenges, and factors influencing uptake in routine kidney care. We applied the i-PARIHS implementation science framework to thematically analyse the data.

**Results:**

Participants reported limited awareness of kidney ePROs and their potential benefits within their communities. Acknowledging constrained resources and competing priorities within the healthcare system, stakeholders suggested harnessing existing regional service improvement networks to support ePRO system implementation into kidney care pathways. They emphasised the need for coordinated efforts among national stakeholder organisations to drive and support local implementation. They also identified high quality evidence of ePROs’ clinical utility and positive impact on kidney care and outcomes as a requirement for successful large-scale implementation, highlighting different types of impact as primarily relevant. Anecdotal evidence of ePRO benefits and real-world evidence of feasibility from local pilots were mentioned as complementary sources of knowledge to strengthen the case for cross-organisational ePRO system implementation in kidney care settings.

**Conclusions:**

Successfully implementing cross-organisational ePRO systems in kidney services requires stakeholders to be better informed about ePROs’ potential value, supported by robust and real-world evidence derived from studies in kidney disease. In a healthcare system under pressure, leveraging existing regional service improvement initiatives and coordinated leadership from a national cross-stakeholder group were considered key drivers of implementation success. Future research should strengthen the evidence base for kidney ePRO systems’ effectiveness and implementation, while also exploring available health IT infrastructure. This will shape implementation strategies that enable healthcare systems to fully realise the potential of ePROs to improve kidney services and outcomes.

**Supplementary Information:**

The online version contains supplementary material available at https://doi.org/10.1186/s41687-026-01137-7.

## Background

Across the globe, collecting patient-reported outcome (PRO) data is increasingly promoted for making health care services more patient-centred and enhancing health outcomes for people with long-term conditions [1–4]. Furthermore, using PRO data for secondary purposes beyond direct care provides opportunities for incorporating the patient perspective into clinical audits, service improvement initiatives, commissioning, and health research [5–8]. However, fully harnessing these benefits requires systems that enable collection of electronic PRO (ePRO) data at a large scale, ideally as part of routine care delivery. In this paper, we will refer to these systems as *ePRO systems*, defined as “ambulatory, non-invasive digital technologies that enable patients to report and record PRO data electronically, remotely and in real-time as part of their usual care, and to transmit this data to healthcare professionals for assessment and clinical decision-making” [9].

ePRO systems enable patient-reported data to flow from a patient-facing system (e.g., smartphone app, patient portal) into the electronic health record system of a healthcare provider (e.g., hospitals). From there, it may be shared with other healthcare providers (e.g., general practitioners, social care organisations) for direct care or with non-healthcare providers (e.g., registries, research organisations) for secondary purposes [9]. This means that large-scale, clinically embedded ePRO systems inherently need to cross organisational boundaries to meet their full potential for improving care and outcomes. This adds considerable complexity to their implementation [10].

Hospital-based services for people living with chronic kidney disease in the United Kingdom (UK) provide an optimal setting for implementing ePRO systems at a national scale. Kidney services are digitally mature compared to other parts of the UK healthcare system, with all kidney providers using an electronic health record system connected to a national infrastructure [10] that facilitates patients’ access and reporting of health and care data [11], as well as data sharing for auditing and research [12]. Moreover, routine collection of patient-reported data has been advocated for enabling patient-centred care in people living with kidney disease [9, 13, 14]. Yet uptake of cross-organisational ePRO systems in UK kidney practice has been slow, with ePRO systems only implemented locally as pilots in the context of research studies [14–19]. This means the potential of ePROs to improve kidney health services and outcomes remains largely unharnessed [20].

Implementing cross-organisational ePRO systems to expedite the scaling up of routine ePRO collection requires the involvement of stakeholders. Stakeholders include patients and healthcare professionals as primary users (i.e., ‘recipients’ or ‘adopters’) of ePRO systems. Although recipients are an essential group, implementation frameworks also highlight the importance of other stakeholder types, such as facilitators (e.g., people or organisations acting as agents to coordinate and support ePRO system implementation into clinical pathways) and leaders (e.g., informal opinion leaders, formal higher-level leaders mandating routine ePRO collection) [21–23]. Involving this wider range of stakeholders and understanding their perspectives and needs enhances implementation success [4, 9, 24, 25], especially considering that what different types of stakeholders expect and want from ePROs may not always align [26]. Some implementation frameworks, such as integrated Promoting Action on Research Implementation in Health Services (i-PARIHS) [22], further encourage explicit analysis of interactions between stakeholders, who can be *recipients* viewing an *innovation* through a particular lens, actors in or influencers of the *context* in which the innovation is implemented, or *facilitators* of the innovation directly (e.g., by sharing their experience to train others) or indirectly (e.g., by using their position to champion an innovation).

Stakeholders acting at this broader level have been largely neglected in studies to date. Several studies in kidney disease [27, 28] as well as other conditions [24, 29–31] explored the perspectives and roles of individual patients and healthcare professionals in mostly local ePRO system implementation. Others reported involving a wider range of stakeholders but in other activities, such as developing a new PRO measure [32], setting a PRO research agenda [33], or shaping ePRO collection in the context of trials [34] or disease registries [7, 35]. This leaves wider stakeholders’ perspectives on how to implement cross-organisational ePRO systems for direct care purposes largely unknown.

Therefore, we explored with wider stakeholders what they considered necessary for implementing ePROs across healthcare organisations to directly support kidney care delivery. Specific objectives were to gain insights into their views on (a) the barriers and opportunities for, (b) the role of stakeholders in, and (c) the types of knowledge needed for successful implementation of cross-organisational ePRO systems in hospital-based services for people living with kidney disease in the UK.

## Methods

We conducted a descriptive qualitative study using semi-structured key informant interviews [36] with representatives of national stakeholder groups relevant to delivery of hospital-based kidney healthcare services in the UK. We reported our study in line with the consolidated criteria for reporting qualitative research (COREQ; see Supplement 1 for the completed checklist) [37].

### Theoretical framework

The i-PARIHS framework guided our study [22]. It consists of four core constructs: innovation (i.e., the change being introduced and the factors relating to it); recipients (i.e., individuals and teams that need to adopt the innovation); context (i.e., the internal and external environment where implementation is taking place); and facilitation (i.e., implementation enablers). The latter construct of facilitation is central to i-PARIHS, with facilitators’ roles involving assessing and integrating the other three constructs. Together, they determine implementation success, which can be defined as achieving the implementation goals as resulting from *“the facilitation of an innovation with the recipients in their (local*,* organisational and health system) context*”.

i-PARIHS and its predecessor (PARIHS) have been used previously to understand and inform strategies [38, 39], such as for implementing person-centred patient participation in kidney care [40] and PRO implementation in other clinical settings [31, 41]. To our knowledge, iPARIHS has not yet been applied to implementing ePRO systems across kidney healthcare organisations. Given the complexity of this type of implementation, the broad range of stakeholders this involves, and the interactions between them, we employed iPARIHS to help us generate recommendations to address these elements.

### Study context

Hospital-based kidney healthcare services in England are delivered by over seventy kidney centres across the country. They provide outpatient care and kidney replacement therapy to people with advanced chronic kidney disease. The UK Kidney Association is the main national professional body, commissioning and coordinating a wide variety of activities, from the national audit (UK Renal Registry) to an annual conference, and from a quality improvement network to developing clinical practice guidelines. There are two national patient charities (Kidney Care UK and the National Kidney Federation) offering patient support and advocacy. They collaborate with professional bodies on patient-important topics, such as the annual survey of patient-reported care experience among over 13,000 kidney patients [13]. Lastly, the NHS Clinical Reference Group for Renal Services advises NHS England on commissioning specialized kidney services [42], developing national policies, quality standards and service specifications to reduce care variation, enhance equitable access, and improve patient outcomes.

### Participant selection and recruitment

We recruited key informants using stratified purposive sampling combined with snowball sampling [43], defining key informants as professionals who represented national stakeholder groups involved in delivering, evaluating, improving, or commissioning hospital-based kidney healthcare services in England (see Box 1).

**Box 1:** Stakeholder groups and the types of organisations representing them

Stakeholder group	Represented by	Example organisation
Healthcare professionals providing kidney care	Professional bodies	UK Kidney Association; Association of Nephrology Nurses
People living with kidney disease	Patient organisations	Kidney Care UK; National Kidney Federation
NHS commissioners	Commissioning bodies	NHS Clinical Reference Group for renal services
Facilitators	Organisations ^a)^ involved auditing and improving kidney healthcare services	UK Renal Registry; Kidney Quality Improvement Partnership

^a)^ Often, these were part of or commissioned by the UK Kidney Association as the national umbrella organisation for kidney care

Professionals were eligible if they were a staff or board member for an organisation or body representing one of the above stakeholder groups and had experience of shaping or delivering PRO initiatives or other system-wide changes in UK kidney healthcare services.

We initially identified potential participants by searching organisations’ websites and via the professional networks of research team members. We invited potential participants through email, provided them with a participation information sheet about the study (including a section on ‘What is the purpose of the research’), gave them the opportunity ask questions, and obtained written informed consent asked from those who decided to take part. When relevant, they could nominate colleagues as further key informants.

### Data collection

The researcher who conducted all interviews was a cis-male post-doctoral researcher (AE) trained in qualitative and participatory (public) health research, with extensive experience of interviewing healthcare professionals and patients in the UK. He was based within an institute focused on digital health and technology research. Although none of the participants were recruited via the interviewer’s professional network, some knew about the project and had established rapport with AE prior to the interview. At the start of each interview, AE shared information about his professional background and interests and encouraged participants to do the same.

All interviews were individual, semi-structured, and conducted remotely via Zoom™, with only the interview and interviewee present. Each participant was interviewed once, with interviews lasting between 25 and 60 min. The interview guide (see Supplement 2) was pilot-tested within the research team and included topics related to participants’ perspectives on benefits and challenges of routinely collecting and using ePROs in kidney healthcare practice; examples of successful and unsuccessful system-wide changes in kidney care services in the UK; factors perceived to affect successful implementation of cross-organisational ePRO systems; and evidence needed to generate wider buy-in (i.e., stakeholder ownership and active commitment) for routinely collecting ePROs. The interviewer took field notes and audio-recorded all interviews. Recordings were transcribed verbatim by a professional secretarial service with experience of transcribing interviews for qualitative research. Data collection was constrained by how many –often time-poor—key informants we were able to recruit and interview within the time available for delivering the research.

### Data analysis

Analysis happened in two stages, beginning with an inductive thematic analysis followed by a secondary framework analysis using the iPARIHS constructs. The first stage aided familiarisation with the full data set and ensured further analysis was open to emergent findings, with AE initially coding the transcripts using Microsoft Excel while taking an inductive open-coding approach guided by the study’s objectives. This process generated an initial thematic coding framework, which three members of the research team (AE, SK, SNVDV) iteratively refined through discussion (see Supplement 3 for the final coding framework). Once all transcripts had been coded with the agreed, final coding framework, we identified overarching themes and refined them over the course of several meetings to consider their accuracy and agree on their interpretation. In the second stage, we took a similar discussion and consensus approach to mapping themes to constructs from the i-PARIHS framework. This enabled us to sense-check our findings against the theoretical framework and translate the themes into theory-informed recommendations for how to implement cross-organisational ePRO systems to facilitate ePRO collection within kidney healthcare services in England. We presented results in line with this approach by focussing on the themes that we derived from the data before summarising how these corresponded to the iPARIHS constructs.

We did not share transcripts or analysis results with individual participants for feedback but instead used our findings as a starting point for a subsequent stakeholder consultation [44]. This consultation consisted of a full-day, online meeting attended by 58 stakeholders across groups, including several of our interviewees (see Supplement 4 for meeting agenda and activities). We then published an open request for written input from the kidney community, resulting in over 100 suggestions by nine different organisations; see [44] for further detail. We used the results from the consultation to triangulate the current study’s findings and implementation recommendations.

## Results

### Participants

We approached 29 health professionals, of whom 18 responded and accepted our invitation for interview. Of those, 15 were recruited via organisations’ websites and the team’s network, with the remaining three being nominated by other interviewees. Table 1 shows that nine, four, three, and two interviewees primarily represented healthcare professionals, people living with kidney disease, facilitators, or commissioners, respectively. Nine participants represented more than one stakeholder group.

**Table 1:**
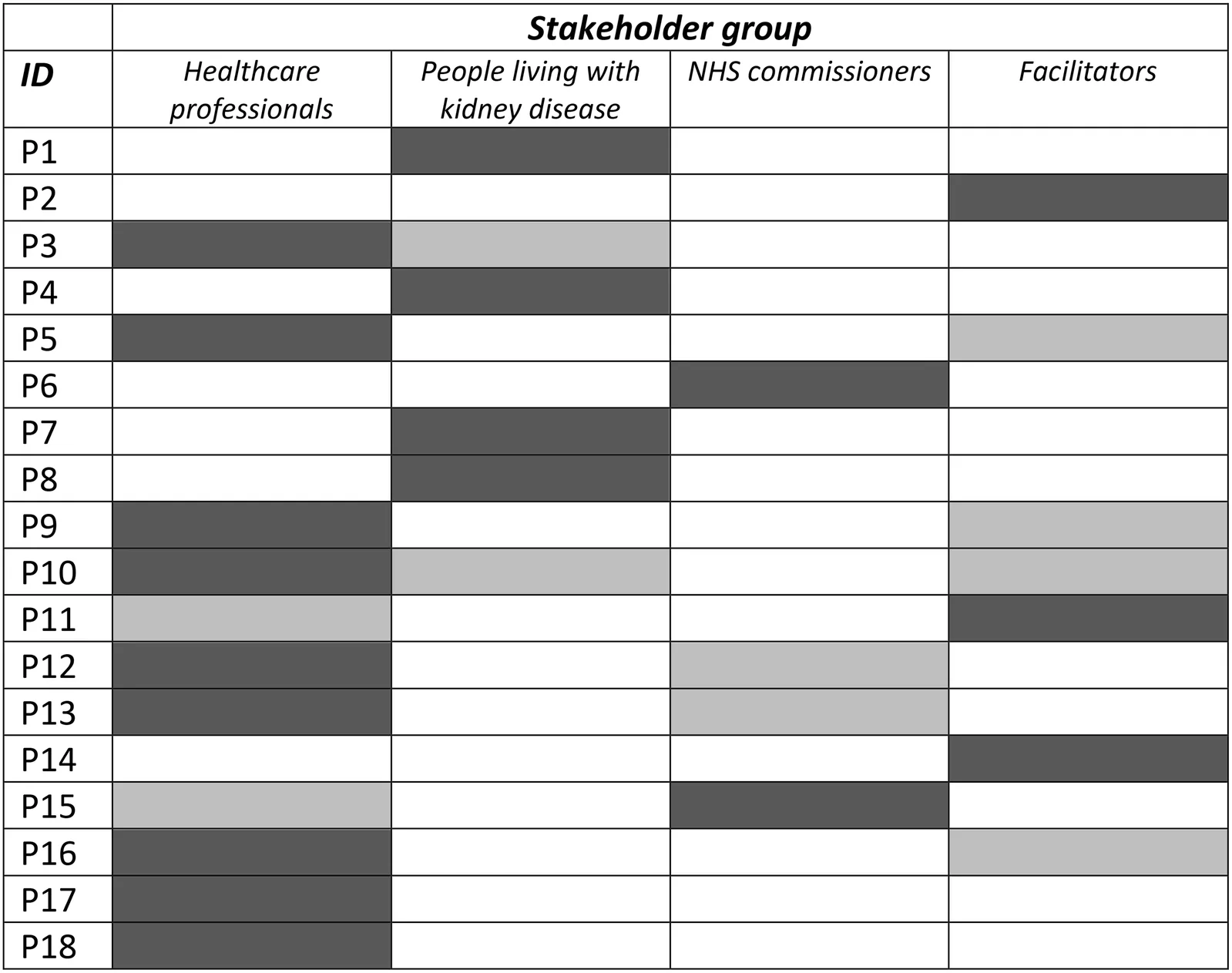
Participant IDs and the stakeholder group(s) they represented

Abbreviations: NHS, National Health Service

(darker and lighter shaded cells indicate participants’ primary and additional affiliations with stakeholder groups, respectively)

### Themes from stakeholder interviews

The following sections describe the themes and recommendations that we derived from the data, illustrated by participant quotes labelled with their ID and primary stakeholder group (see Table 1) and mapped to i-PARIHS constructs (i.e., Recipients, Context, Innovation, and Facilitation). Table 2 presents further quotations relevant to each construct overlaid with the themes and linked to stakeholders’ recommendations for implementing cross-organisational ePRO systems.

**Table 2:** Themes, additional illustrative quotes, and stakeholders’ recommendations mapped to constructs from the i-PARIHS framework

Theme summary	i-PARIHS construct	Additional exemplar quotes ^a)^	Recommendations for implementing cross-organisational ePRO systems
**1. Awareness and beliefs regarding the potential value of ePROs** Not all stakeholders may know the potential value of ePROs, and where they do, they may have different views on how ePROs could lead to improved services and outcomes for people with kidney disease	Recipients	*“It’s still a very emerging concept from my perspective”* (P6, commissioners). “*Not considered as important as clinical outcome measures*.” (P3, healthcare professionals). “*Shift the dialogue between the clinician and the patient in a direction in which it facilitates a better understanding of the association between the underlying medical condition that the patient has and the symptoms that they have associated with that condition that are important for them*.” (P8, people living with kidney disease) *“Flips things around*,* it starts to erode that*,* sort of*,* paternalistic approach to healthcare. It involves the patient to lead on their care*,* potentially*,* if they have the knowledge*,* skills*,* and confidence.”* (P11, facilitators)	Organise an educational or awareness-raising campaign that acknowledges different views on how ePRO data may contribute to enhanced kidney services and outcomes
**2. National support to encourage local buy-in** Stakeholders emphasised the need for local support from staff and patients to implement ePRO systems in an environment with competing priorities and finite resources. National bodies could use their influence to encourage this local support.	Context	**“***So leaders need to be present through all layers of the system*,* top leaders need to set their vision*,* they need to communicate that vision and that could come from anywhere and it could be doctors*,* nurses*,* treatment technicians or patients or anyone else …followers are important as well. So*,* you know*,* it’s not a hierarchy where the leader is better than the followers*,* because the guys who have to implement it*,* who actually do it*,* they’re just as important*” (P13, healthcare professionals) “*Yeah*,* I guess you have to think through*,* who are the people of influence within the organisation actively*,* both formal and informal influence? That’s how we approach it.”* (P2, facilitators) ***“****I think the professional body is the key people… if the professional bodies want it to happen …”* (P14, facilitators)	Establish multidisciplinary groups of local opinion leaders to drive implementation of an ePRO system in their own setting, backed up by national leaders and priorities
**3. Types of evidence and knowledge** Stakeholders suggested that –in addition to evidence of benefit—examples of the usefulness and feasibility of ePROs would support the case for implementing cross-organisational ePRO systems	Innovation	*“Healthcare professional stories as well. If it’s being used in a unit*,* how is it going*,* all of those different things.”* (P16, healthcare professionals) *“Strong case studies to support the use for having clinicians who are using [ePROs] and patients who are using [ePROs] that come together to provide exemplars and toolkits to support the use of [ePROs] for clinicians who are not yet using or who disengage.”* (P9, healthcare professionals). *“Rigorous piloting… beta testing presented over a longer period to iron out more issues.”* (P6, commissioners).	• Establish the evidence base for the positive effect of ePROs on services and outcomes for people with kidney disease • Gather case studies of how individual patients and healthcare professionals have used or would like to use ePROs for their kidney care • Conduct and document local ePRO pilots to gather and share learning on the implementation process
**4. Regional and national stakeholder collaboration** A national group could bring together stakeholder groups and provide guidance on large-scale ePRO implementation, while building on existing regional collaborative networks for service improvement.	Facilitation	*“[national guidance should cover] Every aspect of the work that you’re delivering*,* so I guess what the benefit is*,* how the programmes are structured*,* the frequency of collection*,* the physical mechanism for data sharing*,* the output*,* defining maybe what the outputs are*,* the measures of success.”* (P2, facilitators).	• Harness available regional collaborative service improvement networks • Establish a national cross-stakeholder group to provide guidance and direction for achieving cross-organisational ePRO system implementation

Abbreviations: ePROs, electronic patient-reported outcomes; i-PARIHS, integrated-Promoting Action on Research Implementation in Health Services

a) Codes at the end of each quote reflect participant IDs, followed by their primary stakeholder group; also see Table 1

### Theme 1: Awareness and beliefs regarding the potential value of ePROs

Stakeholders considered the awareness of ePROs and their potential value to be limited in their communities and identified this as a fundamental challenge to scaling up routine ePRO collection. They said that ePROs were considered a new source of data that had yet to be embraced by commissioners and healthcare professionals as adding value to currently available clinical information and suggested a concerted educational or awareness raising effort was required to address this. One of the participants stated:

*Massive educational role*,* to ensure that people understand why they’re doing it*,* and what benefits they might gain from it* (P12, healthcare professionals)

Although most stakeholders agreed that ePROs have a role in improving the care experience and health outcomes for people with kidney disease, their views on *how* ePROs could add value differed. This reflects how differences in **Recipients’** perspectives of value need to be understood. For example, some viewed ePROs as an opportunity to obtain a wider perspective on patients’ health beyond biomedical data, while others mentioned enhanced insights into how patients experience their health outside of the clinical context and into symptoms that may affect their quality of life.

*And it’s collecting data other than just phosphates*,* anaemia*,* and that sort of thing*,* so that we have that whole picture*.(P11, facilitators)

*PROMS should be about targeting the 156 hours when someone is not on dialysis*,* then it should be able to give us a window into how someone is living their life and then asking the question*,* what are the things that we have within our gift to maybe alter and improve?* (P13, healthcare professionals)

Others emphasised the potential for facilitating more patient-centred and personalised care by enhancing communication and shared-decision making. Healthcare professional stakeholders and commissioners viewed ePRO data as a source of information for assessing the quality of services or for better understanding patients’ views on quality of care.

*So rather than them becoming one of the 12 patients who came to the clinic*,* it might be a bit more bespoke for them if they’ve had to take the time to do the narrative about the disease in a more formal process.* (P18, healthcare professionals)

*[from a] commissioning perspective it will give us better information on the quality of the services* (P6, commissioners)

A facilitator stakeholder suggested the possibility of using ePROs to inform population health decisions:

*And I think as a whole*,* you don’t only have to think of benefit in the context of patients*,* clinicians*,* allied health professionals*,* all the team that support the patient*,* providers […] I think there’s potentially an element around doing research and understanding and informing decisions that relate to population health*. (P2, facilitators)

A future educational or awareness raising campaign would need to acknowledge these different views on how ePRO collection may contribute to enhanced kidney care and outcomes.

### Theme 2: National support to encourage local buy-in

This second theme illustrates the interaction between local and national **Context**. Stakeholders believed that national professional groups could use their influence and leadership to encourage ePRO implementation at the local level.

*I’d suggest you’d probably focus to start with…top…just to make sure you’ve got buy-in from the senior stakeholders. So*,* that includes people like [name of UK Kidney Association representative].* (P17, healthcare professionals)

Some also suggested that commissioners could mandate ePRO collection by including it in their specification of kidney services, but others believed implementing ePRO collection in kidney care through contracts and service specifications may have low priority because of the limited knowledge of its potential benefit (see Theme 1). Furthermore, several stakeholders brought up that, while commissioners might be increasingly interested in finding new strategies to improve patient experience and health outcomes, it remained unclear how service improvement could be accomplished in the context of multiple, competing priorities and finite resources:

*With all these things*,* you’re going to be adding something to an already busy service*,* and an already busy time. […] The people you’re asking to make the sacrifice in terms of time and effort*,* aren’t going to be committed to it*,* when they start.* (P12, healthcare professionals)

Related to this, stakeholders emphasised that successful implementation of cross-organisational ePRO systems equally relied on buy-in from healthcare providers and professionals within local organisations, with local staff’s perspectives and capacity being key in determining whether a hospital provided an implementation-enabling **Context**.

*So it all depends on certain people within a [hospital] and their attitude to it.* (P7, people living with kidney disease)

A healthcare professional stakeholder who had previously been involved in commissioning said they thought this buy-in was present among local managers of most but not all kidney healthcare service providers:

*But most [clinical directors]*,* I would have thought*,* are on board with these sorts of things*,* although when I was [clinical reference group] chair I do remember that one unit didn’t want to do it* (P12, healthcare professionals)

Related to the need for local buy-in, stakeholders suggested establishing multidisciplinary groups of champions, including both staff and patients, who could drive the implementation of ePROs within their own setting, encouraged and backed up by national organisations and priorities. For example, managers could decide to assign resources to support ePRO implementation in their centre; clinical and administrative staff members could lead on collecting ePROs; and patients could encourage other patients to report their ePROs, as well as engaging healthcare professionals by asking ePRO results to be discussed during clinic visits.

### Theme 3: Types of evidence and knowledge

Regarding the **Innovation** itself, the most prominent theme suggested that most stakeholders believed it was essential to provide further evidence of the impact of ePROs using patient and care outcomes that were important and meaningful to them. For example, commissioner stakeholders mentioned the need for evidence of routine ePRO collection leading to improved healthcare services, while others said healthcare professionals would need to know that ePROs provide meaningful measures of differences between individual patients:

*…does it actually change the patient pathway or the service pathway in any way*,* shape or form*,* other than just collecting a bit more new information.* (P15, commissioners)

*You want to prove that you can measure people’s differences meaningfully.* (P12, healthcare professionals)

Stakeholders across groups recommended capturing exemplary narratives from patients and healthcare professionals who had personal experience of using ePROs for kidney care and could share insights on their usefulness and feasibility. Related to the latter, stakeholders also suggested conducting targeted local pilots to generate and share real-world, practical learning on how routine ePRO collection could be embedded in clinical care, including what challenges to expect and how to address them.

*You start with your patients*,* the patients have been using it*,* how have they benefitted from it*,* can they be a testament to the ePRO so that you can market it to everybody else to say this is how it benefitted my life and this is how it improved my life. And the same with the people*,* any clinicians or units that have been using it and reviewing it*,* how much they thought it benefitted them as clinicians.* (P1, people living with kidney disease)

*We have to do it in those incrementals*,* test it in a few units*,* get the roadmap and then say*,* here you go*,* this is how we can do it.* (P11, facilitators)

### Theme 4: Regional and national stakeholder collaborations

Several interviewees suggested that establishing and implementing cross-organisational ePRO systems for kidney care could build on existing regional networks where stakeholders were already collaborating to improve the quality of the service. These networks would therefore enable **Facilitation** through bringing together key stakeholders and actors with an implementation and improvement focus.

*In the North West of England we run a network of people who are interested in quality improvements*,* and all of the clinical directors signed up to taking on a quality improvement project every year.* (P14, facilitators)

Related to the influence of national bodies mentioned under Theme 2, many stakeholders recommended establishing a national group to foster collaborations across stakeholder groups and provide guidance on how to achieve large-scale ePRO collection. This further suggests a link between **Context** and **Facilitation** to create an environment that leverages national collaborations to foster implementation.

*I would probably […] create a steering group to decide how is the renal community going to move from where it is today to a national [patient-reported outcome] information system* (P8, people living with kidney disease)

## Discussion

### Summary of findings

We explored wider stakeholders’ perspectives on requirements for implementing ePRO systems across kidney healthcare organisations in England (UK). Stakeholders thought that people in their communities were generally unaware of ePROs and their potential value for improving services and outcomes for people living with kidney disease. While they characterised the NHS as a context with many competing priorities and limited resources, they recognised the potential of existing regional service improvement networks to support implementation of cross-organisational ePRO systems into kidney care pathways. Furthermore, they suggested that national stakeholder organisations should join forces and use their influence to encourage and support local stakeholders to implement ePROs in their setting. Lastly, they identified high quality evidence of the clinical utility and positive impact of ePROs in kidney care settings as a requirement for successful large-scale implementation, highlighting different types of impact as primarily relevant. Anecdotal evidence of ePRO benefits and real-world evidence of feasibility from local pilots were mentioned as complementary sources of knowledge to strengthen the case for cross-organisational ePRO system implementation into UK kidney services.

### Relation to other studies

Many of our findings on ePROs for facilitating direct kidney care aligned with wider stakeholder perspectives on implementing PROs to facilitate service evaluation or research in other areas [7, 34]. For example, they mentioned a lack of awareness of potential PRO benefits within stakeholder communities and considered this an important barrier to large-scale implementation. Like our interviewees, they also highlighted differences between stakeholder groups in what such benefits were perceived to be [34], and recommended the use of case studies as complementary knowledge to promote implementation [7]. Similarly, they highlighted top-down governance as an implementation enabler [7, 34], alongside provision of central funding to support local initiatives [7]. This is in keeping with our participants suggesting that ePRO collection could be mandated in commissioning policies. However, our interviewees specifically noted that this would only work if all stakeholders were sufficiently aware of the potential benefits of PROs. This aligns with previous research employing iPARIHS [39], which suggested that better expressing the benefits of an innovation is essential for creating a facilitating implementation context.

Egholm and colleagues shared lessons learned from an ongoing initiative in Denmark to implement a national ePRO system across conditions to inform direct care [4]. In line with our findings, they highlighted the importance of a national cross-stakeholder steering group to guide and support local implementation efforts, and to align the implementation with existing quality improvement initiatives and strategies. An important difference, however, was their emphasis on the importance and complexity of establishing a national information technology (IT) infrastructure to facilitate cross-organisational data collection and sharing; none of our interviewees mentioned this as a requirement, perhaps because they assumed the UK’s existing national kidney audit would be able to facilitate this. In contrast, Egholm et al. did not identify evidence of clinical utility, impact, or feasibility of ePROs as an important factor. One explanation is that their initiative had been mandated from top-down, assuming therefore that this would be less of a requirement. Alternatively, stakeholders in our sample might have been reluctant to consider evidence from effectiveness research conducted in other clinical domains [20, 45, 46] or primary care settings [47] generalisable to hospital-based kidney services. For instance, much of the existing evidence supporting ePRO effectiveness derives from oncology and centres on symptom monitoring during defined treatment periods (e.g., while on chemotherapy) [45, 46]. In such contexts, patients typically report ePROs over a limited timeframe, and clinical responses are largely confined to treatment modification. This model does not readily translate to kidney disease, where ePRO reporting is often lifelong and clinical responses to ePRO results are more heterogeneous.

### Limitations

One limitation was that participants’ willingness to be interviewed might have indicated a generally positive view on ePRO systems, their potential value, and the feasibility of implementing these across healthcare organisations. Eleven potential key informants did not respond to or declined our invitation, and their decision not to take part might reflect different, possibly less optimistic perspectives on the topic, which might therefore remain underrepresented in our findings.

Another limitation was that our recruitment was time constrained. We judged the information power of our data sufficient because of a relatively focused study aim, high sample specificity, and our use of i-PARIHS as an established theoretical framework [48]. Interviews conducted towards the end of the data collection period strengthened previously derived themes but without adding new insights. Yet, a longer recruitment period would have allowed us to interview more representatives per stakeholder group, increasing the chance of identifying diverging or conflicting stakeholder views that could hamper successful ePRO system implementation if unresolved.

### Implications for practice and research

#### Leverage existing local and regional initiatives and buy-in

Our findings indicated that implementation of cross-organisational ePRO systems in kidney services could be strengthened by leveraging existing regional initiatives and buy-in from local stakeholders. This was confirmed during the subsequent stakeholder consultation we organised to triangulate our findings [44]. It resulted in a recommendation that regional commissioners and operational kidney service delivery networks should develop ePRO implementation strategies aligned with regional priorities and resources and supported by national guidance. This is in keeping with previous work highlighting the importance of balancing national aims with regional and local diversity and autonomy in large-scale ePRO or IT system implementations [31, 49, 50]. A comparable approach was described by Terner et al. in the context of national ePRO implementation for hip and knee surgery in Canada, where national data standards were established to facilitate regional implementation efforts [51].

#### Establish a national cross-stakeholder group to coordinate and drive implementation

Key informants in our study suggested to establish a national group to coordinate and drive cross-organisational ePRO system implementation for UK kidney healthcare services. The need for such a national group was confirmed by other studies [4, 7] and during the subsequent stakeholder consultation [44]. In line with previous suggestions [9, 35], the group might include the stakeholder types represented in our study, complemented by representatives from primary care settings, companies supplying IT systems for kidney services, funders of kidney research, and ePRO experts. They could build on available guidance and instruments to shape their plans and activities (e.g., [25, 52, 53]), while taking forward some of the recommendations suggested in the current study, such as organising a national campaign to raise awareness about ePROs and their potential benefits for kidney care and outcomes.

#### Strengthen the evidence base for kidney ePRO system effectiveness and implementation

Building on our interview and stakeholder group findings and those of previous studies [54, 55], future research should generate robust evidence regarding the (cost-) effectiveness of kidney ePRO systems, as well as strategies to optimise their implementation into kidney care pathways. A multicentre, pragmatic non-inferiority trial conducted across three outpatient clinics in Denmark compared the impact of PRO-based, PRO-telephone follow-up, and usual care on changes in kidney function but yielded inconclusive results [56]. Other trials, some of which are ongoing, focused exclusively on in-centre haemodialysis settings [57–59], indicating a need for future studies targeting broader kidney populations. These should build on ePRO research from other areas [20, 45, 47] and ideally combine elements of comparative-effectiveness and implementation research to strengthen the evidence base for ePRO systems as well as facilitate their successful implementation into routine clinical care [60, 61].

#### Explore capabilities of available IT infrastructures to support ePRO system implementation

An unanswered question from our study concerns the specific IT requirements for enabling successful implementation of ePRO systems across organisational boundaries for kidney care as well as in other clinical contexts. Stakeholders in our subsequent consultation [44], as well as others [4, 31, 52, 53, 62] consistently identified this as a critical component of large-scale ePRO implementation. Core requirements included the ability to integrate with existing electronic health records and patient portals, intuitive and accessible user interfaces, and reporting ePRO results to patients and healthcare professionals in meaningful and actionable formats.

Future research may therefore systematically explore available IT systems that underpin UK kidney services at local, regional and national levels. For example, by interviewing a focused sample of technical stakeholders, such as local and national IT staff or suppliers of electronic health record systems and patient portals. Building on the approach taken by Anderson et al. [14], such interviews might focus on identifying opportunities and gaps in current IT infrastructures by assessing the systems’ capabilities to collect, report, and manage ePRO data, and mapping these against stakeholder requirements. In turn, this could inform the development of technically feasible and user-centred strategies for cross-organisational ePRO system implementation.

## Conclusion

To support successful implementation of ePRO systems across healthcare organisations in UK kidney services, stakeholders need greater awareness of their potential benefits, informed by both robust evidence and real-world examples demonstrating feasibility and effectiveness. In a resource-constrained healthcare system with competing priorities, leveraging existing regional service improvement initiatives and coordinated leadership from a national cross-stakeholder group are considered key drivers of implementation success. Future research should strengthen the evidence base for the effectiveness and implementation of ePRO systems in kidney services, while also further exploring opportunities and gaps in available health IT infrastructure. This will help shape implementation strategies that enable healthcare systems to fully realise the potential of ePROs to improve kidney services and outcomes.

## Supplementary Information

Below is the link to the electronic supplementary material.

Supplementary Material 1

## Data Availability

The interview transcripts are not available for sharing as we did not obtain individuals’ consent for this. Summary materials from the qualitative analysis are available from the corresponding author on request.

## Abbreviations

CKD: Chronic kidney disease
COREQ: Consolidated criteria for reporting qualitative research
ePRO: Electronic patient reported outcome
i-PARIHS: integrated-Promoting Action on Research Implementation in Health Services
NHS: National Health Service
PRO: Patient-reported outcome
UK: United Kingdom
