# Supplementary material for: Wider stakeholders’ perspectives on successfully implementing electronic patient-reported outcome systems across organisations: a qualitative study in kidney care
Source: J Patient Rep Outcomes. 2026 Jul 15;10:187. doi: 10.1186/s41687-026-01137-7 (PMC13658655; doi:10.1186/s41687-026-01137-7)
Supplement: Supplementary file 1 [file 41687_2026_1137_MOESM1_ESM.pdf]

Supplementary materials

Supplement 1: Completed consolidated criteria for reporting qualitative studies (COREQ) checklist

No. Item	Guide questions/description	Reported on Page #
**Domain 1: Research team and reflexivity**		
Personal Characteristics		
1. Inter viewer/facilitator	Which author/s conducted the inter view or focus group?	7
2. Credentials	What were the researcher's credentials? E.g. PhD, MD	7
3. Occupation	What was their occupation at the time of the study?	7
4. Gender	Was the researcher male or female?	7
5. Experience and training	What experience or training did the researcher have?	7
Relationship with participants		
6. Relationship established	Was a relationship established prior to study commencement?	7
7. Participant knowledge of the interviewer	What did the participants know about the researcher? e.g. personal goals, reasons for doing the research	7,8
8. Interviewer characteristics	What characteristics were reported about the inter viewer/facilitator? e.g. Bias, assumptions, reasons and interests in the research topic	7,8
**Domain 2: study design**		
Theoretical framework		
9. Methodological orientation and Theory	What methodological orientation was stated to underpin the study? e.g. grounded theory, discourse analysis, ethnography, phenomenology, content analysis	5
Participant selection		
10. Sampling	How were participants selected? e.g. purposive, convenience, consecutive, snowball	7
11. Method of approach	How were participants approached? e.g. face-to-face, telephone, mail, email	7
12. Sample size	How many participants were in the study?	9
13. Non-participation	How many people refused to participate or dropped out? Reasons?	9,16
Setting		
14. Setting of data collection	Where was the data collected? e.g. home, clinic, workplace	8
15. Presence of non-participants	Was anyone else present besides the participants and researchers?	8
16. Description of sample	What are the important characteristics of the sample? e.g. demographic data, date	9,Table 1
Data collection		
17. Interview guide	Were questions, prompts, guides provided by the authors? Was it pilot tested?	8,Suppl 2
18. Repeat interviews	Were repeat interviews carried out? If yes, how many?	8
19. Audio/visual recording	Did the research use audio or visual recording to collect the data?	8
20. Field notes	Were field notes made during and/or after the interview or focus group?	8
21. Duration	What was the duration of the inter views or focus group?	8
22. Data saturation	Was data saturation discussed?	8,16

Supplement 1 cont.

23. Transcripts returned	Were transcripts returned to participants for comment and/or correction?	9
**Domain 3: analysis and findings**		
Data analysis		
24. Number of data coders	How many data coders coded the data?	8
25. Description of the coding tree	Did authors provide a description of the coding tree?	Suppl 3
26. Derivation of themes	Were themes identified in advance or derived from the data?	8
27. Software	What software, if applicable, was used to manage the data?	8
28. Participant checking	Did participants provide feedback on the findings?	9
Reporting		
29. Quotations presented	Were participant quotations presented to illustrate the themes/findings? Was each quotation identified? e.g. participant number	9-14,Table 2
30. Data and findings consistent	Was there consistency between the data presented and the findings?	9-14,Table 2
31. Clarity of major themes	Were major themes clearly presented in the findings?	9-14,Table 2
32. Clarity of minor themes	Is there a description of diverse cases or discussion of minor themes?	9-14,Table 2

Supplement 2: Interview topic guide

- Can you introduce yourself and explain what your role is with regard to kidney services in the UK?
- What do you know about patient-reported outcomes, in general and from professional/personal experience?
    - What is your perception of where we are in the UK in terms of collecting/using patient-reported outcomes in people with chronic kidney disease?
- Do you think routinely collecting and using patient-reported outcome data as part of kidney services is a good idea, both in general and for you as a [stakeholder type]/your organisation in particular? Why (not)?
    - How could routinely collected patient-reported outcome data be used, by the renal service as a whole and by you as a [stakeholder type]?
    - What do you see as the main benefits?
    - What do you see as potential risks and challenges?
    - Do you think there are additional or different risks and benefits of collecting patient-reported outcome data electronically?

It requires a system-wide change to go from where we are now to a situation where all kidney units are routinely collecting and using electronic patient-reported outcomes (or ePROs) in all their patients.

- Can you think of a recent example of a successful system-wide change in kidney services in the UK? E.g. substantial revision of the tariff system, introduction of a new service or technology, a change in how an existing service was being delivered, etc.
    - Can you describe what the change process was like from your perspective?
    - Who played a key role in the process and how (e.g. stakeholder groups, decision makers, opinion leaders)? What was your role/the role of your organisation?
    - In your opinion, what were the main factors that made this a successful change? What were the main barriers and how were they addressed?
- Similarly, can you think of a recent example of a system-wide change in UK kidney services that was not successful?
    - Can you describe what the change process was like from your perspective?

  - Who played a key role in the process and how (e.g. stakeholder groups, decision makers, opinion leaders)? What was your role/the role of your organisation?
  - In your opinion, what were the main factors that impeded the change from happening?

Now thinking of introducing routine collection and use of ePROs as part of kidney services in the UK.

- Who would you consider playing a key role in making this system-change wide change a success? (e.g. stakeholder groups, decision makers, opinion leaders)?
  - What is your perception of how (un)supportive these key players will be of UK-wide ePRO collection? Do you have any suggestions for how these potentially unsupportive players could be convinced?
- What will be the main facilitators for making this a successful change? What may be the main barriers and how could they be addressed?
  - Are there any financial and regulatory requirements for making the change possible? If so, to what extent are these already in place?
- How do you see your role as a [stakeholder type] in supporting UK-wide routine collection of ePROs in kidney services?
  - What would you need to fulfil this role?
  - Are there any specific local or national incentives or disincentives for you to fulfil this role?
- What evidence would you need to support routine collection of ePROs as part of kidney services (e.g., randomised trials that evaluate the impact of ePRO collection on patient outcomes, health economics evaluations, compelling case studies)?

- Is there anything else you would like to add that we have not yet discussed?

Supplement 3: Stakeholder coding framework (final version)

Stakeholder background

Value of PRO data

- Possible uses of PROs
- Limitations of PROs

Collecting and using ePRO data

- Challenges with collecting ePRO data
- Influencing factors for collecting ePRO data
- Patient barriers to completing ePROMs

Successful programme implementation

- Example of successful programme
- Resources for successful programme implementation
- Factors influencing successful programme implementation
- Stakeholders' role in successful programme implementation
- Limitations of successful programme implementation

Unsuccessful programme implementation

- Example of unsuccessful programme
- Resources for unsuccessful programme implementation
- Factors influencing unsuccessful programme implementation
- Barriers/challenges for successful implementation

Nationwide ePRO implementation

- Evidence needed for implementation
- Factors to consider with nationwide ePRO implementation
- Barriers to implementing a nationwide ePRO programme
- Key stakeholders and their role in nationwide ePRO implementation

System context

Health care professionals using ePROs

- Health care professionals' experience with ePROs
- Factors/ strategies to consider for using ePRO data
- Challenges with incorporating ePRO data into practice
- Strategies to encourage health care professionals to use ePROs

Supplement 4: Agenda & Activities for full day, online meeting (n=58 attendees) as part of the stakeholder consultation

**Start**	**Item**	**Session lead**
09:30-10:00	**Welcome: summit aims and introductions**<br>Introduction by organisers, welcome videos by RA/BRS/NKF, and presentation by patient (KCUK) representative	**[chair of UKKA]**
10:00-11:00	**Plenary session: the current ePRO landscape**<br>*(two 20-minute pre-recorded talks, followed by Q&A)*<br>• Local/national ePRO implementation in oncology (UK). Prof Galina Velikova, University of Leeds, UK.<br>• National implementation of ePROs in Australia and New Zealand. Dr Rachael Morton, University of Sydney, Sydney, Australia	**[summit co-host]**
11:00-11:30	COFFEE BREAK	
11:30-12:30	**Breakout session 1: current barriers and challenges**<br>1. ePRO Measurement– Derek Kyte/Ben Fletcher<br>2. IT Infrastructure – Sabine van der Veer/Afzal Chaudhry<br>3. Implementation – Sarah Knowles/Rachel Gair<br>4. ePRO-generated Inequalities – Angelo Ercia/Shivani Sharma<br>5. ePROs in multimorbidity – Simon Fraser/Stewart Mercer	**[moderators]**
12:30-13:30	LUNCH	
13:30-14:30	**Breakout session 2: future opportunities and potential solutions**<br>1. ePRO Measurement– Derek Kyte/Ben Fletcher<br>2. IT Infrastructure – Sabine van der Veer/Afzal Chaudhry<br>3. Implementation – Sarah Knowles/Rachel Gair<br>4. ePRO-generated Inequalities – Angelo Ercia/Shivani Sharma<br>5. ePROs in multimorbidity – Simon Fraser/Stewart Mercer	**[moderators]**
14:30-15:00	COFFEE BREAK	
15:00-16:00	**Whole group feedback/discussion**<br>• Summary of breakout group discussions<br>• Identifying priorities of challenges coming out of breakout sessions 1	**[external facilitator with patient representative]**
16.00-16:15	**Summary and next steps**<br>How we will take the results of the summit forward through the development of a roadmap, in consultation with summit attendees	**[summit co-hosts]**
**16:15**	**SUMMIT CLOSE**	
